# Dynamics and manipulation of ferroelectric domain walls in bismuth ferrite thin films

**DOI:** 10.1093/nsr/nwz176

**Published:** 2019-11-08

**Authors:** Shuyu Xiao, Yaming Jin, Xiaomei Lu, Sang-Wook Cheong, Jiangyu Li, Yang Li, Fengzhen Huang, Jinsong Zhu

**Affiliations:** 1 National Laboratory of Solid State Microstructures and Physics School, Nanjing University, Nanjing 210093, China; 2 Collaborative Innovation Center of Advanced Microstructures, Nanjing University, Nanjing 210093, China; 3 Rutgers Center for Emergent Materials and Department of Physics and Astronomy, Rutgers University, Piscataway, NJ 08854, USA; 4 Shenzhen Key Laboratory of Nanobiomechanics, Shenzhen Institutes of Advanced Technology, Chinese Academy of Sciences, Shenzhen 518055, China; 5 Department of Mechanical Engineering, University of Washington, Seattle, WA 98195, USA

**Keywords:** domain wall, ferroelectric, thin film, piezoresponse force microscopy, dynamics

## Abstract

Ferroelectric domain walls differ from domains not only in their crystalline and discrete symmetry, but also in their electronic, magnetic, and mechanical properties. Although domain walls provide a degree of freedom to regulate the physical properties at the nanoscale, the relatively lower controllability prevents their practical applications in nano-devices. In this work, with the advantages of 3D domain configuration detection based on piezoresponse force microscopy, we find that the mobility of three types of domain walls (tail-to-tail, head-to-tail, head-to-head) in (001) BiFeO_3_ films varies with the applied electrical field. Under low voltages, head-to-tail domain walls are more mobile than other domain walls, while, under high voltages, tail-to-tail domain walls become rather active and possess relatively long average lengths. This is due to the high nucleation energy and relatively low growth energy for charged domain walls. Finally, we demonstrate the manipulation of domain walls through successive electric writings, resulting in well-aligned conduction paths as designed, paving the way for their application in advanced spintronic, memory and communication nano-devices.

## INTRODUCTION

Domain walls (DWs) in ferroic materials serve as active elements possessing dramatic mechanical, electronic, optical, and magnetic properties aside from ferroic domains, offering great potential for technological applications in spintronics, memory devices, and communications [1,2]. As acquiring stable functional DWs is a precondition for potential applications, understanding DW dynamics and developing DW manipulation approaches in ferroics are of great fundamental and practical importance. Up to now, DW dynamics have been studied in various ferromagnetic [3–8] and ferroelectric [9–17] materials. For ferroelectric DWs, it is well known that external stimuli, such as electric fields [9], mechanical strain [9,10], and temperature [11–13], could manipulate DW morphology and stability. Besides, the DW movement could also be affected by inertial properties of the sample, e.g. periodic potential in lattice structures could lead to oscillations in DW motion under a dc electric field [14], and the exponential coefficient in Merz's law decreases with the increase of sample thickness and domain sizes [15–17]. Moreover, intrinsic characteristics of DWs could play an important role as well, e.g. DW velocity in SmI is several times larger parallel to smectic layers than in the perpendicular direction [18], and it depends inversely on the domain radius [1]. One of the foremost characteristics of DWs is bound charges [19–23], the impact of which in DW dynamics is mostly investigated in theoretical calculations [24,25]. This work aims at providing experimental insight into DW dynamics of differently charged DWs under electric fields.

Here we study the subject in BiFeO_3_ (BFO) films, which are an attractive platform to study DW dynamics and properties, taking advantages of their regular DW shapes as compared to other improper ferroelectrics, such as YMnO_3_, ErMnO_3_ and TbMnO_3_ [26–30]. Additionally, BFO exhibits up to eight polarization directions (along the <111> directions), providing relatively more switchable states than traditional ferroelectrics such as LiNbO_3_, PZT and BaTiO_3_ [10,15,24,31–33]. (001)-oriented BFO films with mosaic domain structure are chosen as our samples, which supply complicated DW types, i.e. differently charged DWs in various lengths and directions, unlike stripe domain BFO with mostly long straight head-to-tail DWs. Our previous work discovered that three differently charged DWs with the same DW angle show considerable difference in conductivity [21]. To further improve the tunabilities in the functional DWs, understanding the switching dynamics of differently charged DWs is crucial.

In this work, using piezoresponse force microscopy (PFM), the dynamics of three types of DWs (head-to-head (H–H), head-to-tail (H–T), and tail-to-tail (T–T)) in (001) epitaxial BFO films are found to vary with applied electric bias. This phenomenon is attributed to the pinning effect of charged defects, partly supported by high-resolution X-ray photoelectron spectroscopy (HR-XPS). Beyond the investigations, a two-step scanning scheme corresponding to low and high voltages, respectively, was designed and carried out in a trial experiment, demonstrating the controllable feature of conductive paths in systems with complex domain configurations. Our results establish better understanding of DW dynamics, shedding light on potential DW nanoelectronics based on ferroelectrics.

## RESULTS

Epitaxial 30 nm-thick BFO films were fabricated on (001)-oriented SrTiO_3_ single-crystal substrates using pulsed laser deposition, with a 40 nm-thick bottom electrode of epitaxial SrRuO_3_ (SRO) for the purpose of electrical contact. The out-of-plane (OP) polarizations are downward-pointing, and most DWs are of 71° in the as-grown BFO films. The 3D polarization configuration is achieved by composing PFM images of three mutually perpendicular directions (*x*-in-plane (*x*-IP), *y*-IP, OP) on a commercial scanning probe microscope (SPM) (Dimension Icon, Bruker) (see details in Supplementary Data, Part A) with platinum–iridium-coated conductive tips (SCM-PIT, Bruker) [20,21,34]. During PFM detection, ac voltage of 4 V with 25 kHz and a scan rate of ∼0.5 Hz were used. The dc voltage was applied to the bottom electrode and the scan rate was 0.5 Hz in the polar scan processes. Conductive atomic force microscope (cAFM) images were measured with 4 V applied between the bottom electrode and the grounded tip (SCM-PIT), after a pre-poling process with applied voltage no less than 4 V, to ensure the stability of cAFM images where the switching current is excluded. The high-resolution X-ray photoelectron spectroscopy (HR-XPS) was measured at room temperature on a thermo Scientific K-Alpha system, equipped with a monochromatized Al Kα X-ray source of 1486.6 eV. The standard deviation of the XPS peak position is about 16 meV, which is smaller than the step size of 50 meV used in our experiments.

**Figure 1.**
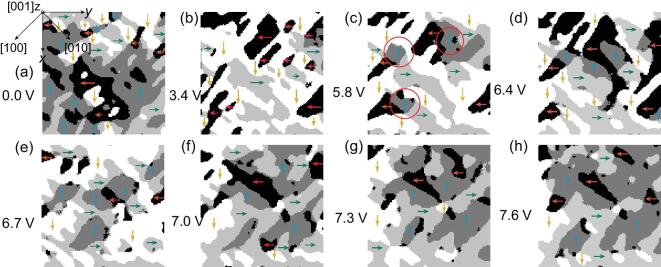
IP domain structure of (001) BFO/SRO/STO under various applied voltages: (a) 0.0 V, (b) 3.4 V, (c) 5.8 V, (d) 6.4 V, (e) 6.7 V, (f) 7.0 V, (g) 7.3 V and (h) 7.6 V. The crystal axis and the experimental coordinate are depicted in the top-left corner. IP polarizations are marked by colored arrows: −*x* (blue), +*x* (yellow), −*y* (red), +*y* (green). OP polarizations are [$00\bar{1}$] for 0 V and [$001$] for other voltages in the whole area depicted.

As shown in Fig. 1a–h, the 3D polarization configurations composed from PFM images (see Supplementary Data, Part B) of a $2{\rm{\ \mu }}{{\rm{m}}} \times 2{\rm{\ \mu }}{{\rm{m}}}$ area after polar scanning with various electrical biases ranging from 0 V to 7.6 V were obtained. The IP polarizations of the –*x*, +*x*, −*y*, +*y* directions are indicated by blue, yellow, orange and green arrows, respectively. The OP polarizations in the whole area of Fig. 1 change from the −*z* to the +*z* direction, as 3.4 V is applied to the bottom electrode (see Supplementary Data, Part C), and they stay the same as the applied voltage ranging from 3.4 V to 7.6 V. The IP polarization develops and the domain configuration reorganizes with the increase of bias voltage (Fig. 1b–h), while a sudden change happens at 6.7 V.

As mentioned above, 71° DWs dominate in the as-grown (001) BFO thin film. This feature remains unchanged after the polarization switching and the domain reconfiguration caused by the electric poling. Notably, 71° DWs can be categorized by the bound charges on them, i.e. H–H, H–T and T–T DWs, which possess different conductivities [21–23,27,35,36]. Tuning these DWs relies on the understanding of their properties and dynamics under different conditions.

**Figure 2.**
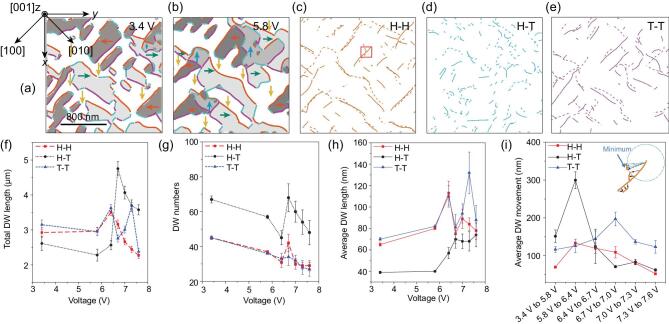
Domain patterns after 3.4 V (a) and 5.8 V (b) poling. Dark, white, light-gray, and dark-gray areas represent domains with polarizations along [$1\bar{1}1$], [$111$], [$\bar{1}11$], and [$\bar{1}\bar{1}1$], respectively. H–H, H–T and T–T DWs are colored orange, light blue and purple, respectively. (c) H–H, (d) H–T, and (e) T–T DW movements before and after 5.8 V poling. DWs before and after poling in (c–e) are depicted as solid and dashed lines, respectively. (f) Total lengths, (g) numbers and (h) average lengths of DWs under various voltages. (i) Average DW movement during each poling process. Inset of (i) sketches how DW movement is measured. Solid and dashed orange lines are H–H DWs before and after 5.8 V poling, respectively, enlarged from the red square in (c).

Here we distinguish three kinds of DWs with different charge conditions from Fig. 1. For example, domain configurations under 3.4 V and 5.8 V are depicted in Fig. 2a and b, with arrows indicating the IP polarization directions, and colored lines indicating differently charged DWs, i.e. H–H, H–T and T–T DWs, marked in orange, blue and purple, respectively. The DW movements from 3.4 V to 5.8 V are displayed in Fig. 2c–e, with solid and dashed lines indicating DWs before and after the higher voltage is applied, respectively. (See another example in Supplementary Data, Part D).

As shown in Fig. 2f–i, the dynamics and related characteristics of the three kinds of DWs under voltages from 0 V to 7.6 V are obtained statistically. Several key features need to be highlighted: (i) Total

DW length. This is the sum of DW lengths, classified by DW types (H–H, H–T, T–T), indicating preferred DW types in the system. As shown in Fig. 2f, charged (H–H and T–T) DWs dominate in the low-voltage region (3.4–6.4 V). When the bias voltage increases to 6.7 V, the total length of uncharged (H–T) DWs increases strikingly. In the high-voltage region (7.0–7.6 V), the total length of uncharged DWs is obviously larger than that of other DWs. (ii) DW numbers and average DW length. The variation of DW numbers (numbers of separate DWs) for three types of DWs with voltage is almost the same (Fig. 2g): DW numbers decrease in general with increasing bias voltage, while there is a peak at 6.7 V. The average DW length is the total DW length divided by the DW numbers. As shown in Fig. 2h, the average DW lengths show a roughly increasing trend with the increase in bias voltage, coincident with the increase in domain sizes. Compared with uncharged DWs, the charged DWs possess fewer numbers but longer average lengths, which is reasonable if we consider the high energy needed to create charged DWs and the relatively lower energy for growth. Notably, the average length of T–T DWs is obviously longer than that of others in the high-voltage region (Fig. 2h). (iii) Average DW movement. Taking the enlarged picture of H–H DWs in Fig. 2c as an example (inset of Fig. 2i), we define the shortest distance between one point in the solid-line DW and its neighboring dashed-line DW as the movement of the point in the solid-line DW. The summed-up movements of all data points in solid-line DWs in Fig. 2c divided by the data point number is the average DW movement of H–H DWs when the poling voltage increased from 3.4 V to 5.8 V, indicating the DW mobility. Statistical results are displayed in Fig. 2i. The average movements of the three kinds of DWs first increases and then decreases with increasing bias voltage. It is also interesting to note that, in the high-voltage range, the average movement of T–T DWs is obviously larger than that of the H–H and H–T DWs.

**Figure 3.**
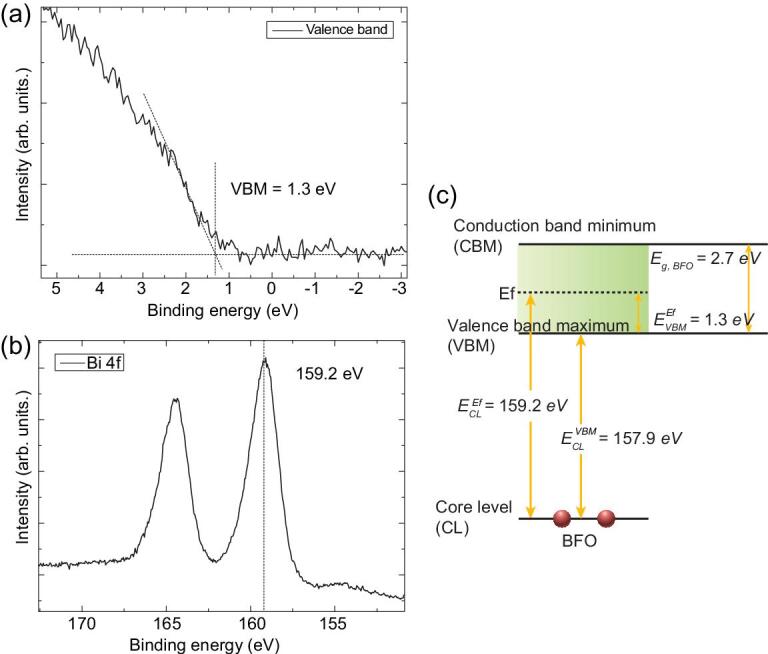
High-resolution X-ray photoelectron spectroscopy (XPS) of BFO films. (a) XPS spectrum near the Fermi level. (b) Bi 4f core level XPS spectrum. (c) Sketch of the Fermi-level calculation from the core lines of Bi 4f.

The above statistics reveals the clear difference of DW dynamics among the three types of DWs. Generally speaking, in the voltage range studied, the H–H and T–T DWs are fewer in number (Fig. 2g), while presenting some advantages in average DW length (Fig. 2h). This behavior might benefit potential DWs from the application point of view, considering that charged DWs are usually related to high conductivity. Additionally, the average movement of H–H DWs is relatively small in the whole voltage range, indicating lower mobility. To understand this, the energy band alignment was examined by HR-XPS (Fig. 3). The Fermi level of the sample is 1.3 eV above the valence band maximum (Fig. 3a), which is consistent with what can be determined from the core lines of Bi 4f (Fig. 3b and c). Note that 1.3 eV is smaller than half the value of the band gap of perfect BFO (∼2.7 eV) [1,37]. This reveals that our BFO film is slightly p-type, i.e. there are more Bi vacancies than O vacancies, and more holes than electrons.

In BFO thin films, negatively charged Bi vacancies and positively charged O vacancies accumulate around H–H and T–T DWs, respectively [38]. They could increase the energy of charged DWs for nucleation and transverse movement, while decreasing the energy for longitudinal growth, thus resulting in lower DW numbers (Fig. 2g) and new DW numbers (see Supplementary Data, Part E), compensated by the enlargement of the average DW length. It is noteworthy that the abundant Bi vacancies in our p-type sample gathering around H–H DWs would greatly reduce their mobility. Under high voltages, the electrical energy could effectively drive T–T DWs, leading to their relatively higher mobility and longer average length compared with other DWs. Additionally, holes, the dominating carriers in the sample, would also gather around T–T DWs, which is a critical reason for the high conductivity compared with other DWs, as previously reported [21,39,40].

In addition to the diverse mobility of the three kinds of DWs, we find an interesting role of IP electric field from the scanning cantilever on the polarization switching and the domain reconfiguration. Although OP electric field dominates the poling process, the IP field is particular for the scanning probe microscopy while non-negligible for the polarization control.

**Figure 4.**
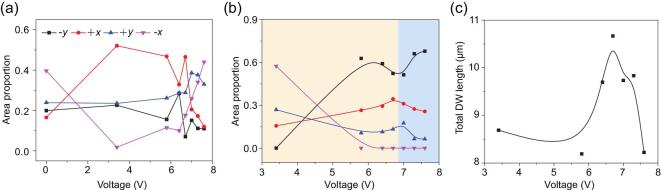
(a) Domain area proportion of four IP polarization directions in the area of Fig. 1 under voltages from 0 V to 7.6 V. (b) Area proportion of four switching angles during the poling process in the area of Fig. 1 under voltages from 3.4 V to 7.6 V. (c) Total DW length in the area of Fig. 1 under voltages from 3.4 V to 7.6 V.

As shown in Fig. 4a, in the as-grown film [0 V], the domain area with −*x*-IP polarization is obviously larger than other domains, possibly due to the substrate and the preparation conditions [41]. Then, at 3.4 V, accompanied by the dominant OP electric field-caused 180° switching [42,43] (Fig. 4b), the area proportions of −*x*- and +*x-*IP polarization reverse (Fig. 4a). When the bias voltage increases to 5.8 V, the IP electric field along the slow scan direction (−*x*) starts to exert an influence [19,28,44]. Along with the emergence of new domains with −*x*-IP polarization (red circles in Fig. 1c), the area ratio of +*x*-IP polarization decreases slightly while that of −*x*-polarization increases (Fig. 4a). At the same time, the proportion of 71° switching becomes higher than 109° and 180° switching (Fig. 4b). As the poling voltage increases to 6.4 V and 6.7 V, the domain configuration changes significantly (Fig. 1d and e). The nucleation and growth of new domains lead to the increase of the total DW length (Fig. 4c), reaching a maximum at 6.7 V, accompanied by a decrease of average domain size (Fig. 1e). With further increase of the poling voltage to above 6.7 V, the combined impact of IP electric field from both the slow scan direction (−*x*) and the cantilever (+*y*) results in the constant enlargement of the −*x* and +*y* domains (Fig. 4a), as well as a gradual decrease of total DW length (Fig. 3c), as large blocks of domains could be observed at 7.6 V (Fig. 1h).

From the above discussions, we note that, under higher poling voltages with 6.7 V as the turning point, on one hand, conductive T–T DWs exhibit higher mobility and longer average DW length; on the other hand, IP electric field become more influential so as to make larger domains with preferred polarization directions. Thus, it is reasonable and technically feasible to produce T–T DWs as conducting paths among different kinds of DWs in p-type BFO.

**Figure 5.**
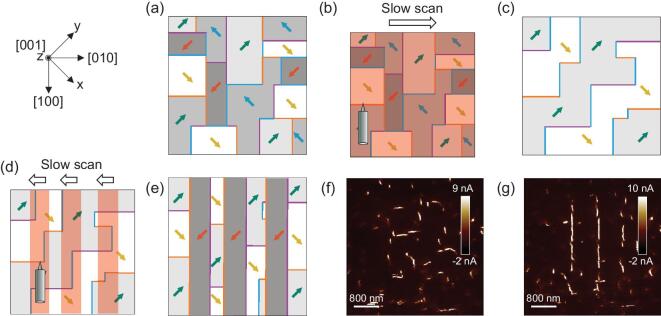
Schematics (a–e) and experimental results (f, g) of conduction path manipulation process. (a) Initial chaotic domain configuration. (b) Low-voltage (4 V) poling scan in the red box. (c) Domain configuration after low-voltage scan. (d) High-voltage (8 V) poling scan in red boxes. (e) Domain configuration after high-voltage scan. IP polarization directions are indicated by colored arrows, and H–H, H–T and T–T DWs are marked by orange, blue and purple lines, respectively, in (a–e). Experimental cAFM current mapping indicates conductive T–T DWs (bright lines) of the initial state (f) and the final state (g).

To demonstrate this idea, we present a two-step poling scheme in an area with chaotic initial domain configuration (Fig. 5a). Though partly broken or curved, DWs in epitaxial BFO films align mostly along the [100] and [010] directions in the surface plane. For simplicity, we illustrate ideal DWs as long and straight. The experimental coordinate of Fig. 5 is $45^\circ $ rotated from that of Figs 1 and 2. (i) Low-voltage poling. As depicted in Fig. 5b, the whole target area is repeatedly polar-scanned with 4 V and 0 V applied at the bottom electrode and tip, respectively, with the slow scan direction fixed to [$010$]. As the electrical field is pointing from the bottom electrode towards the SPM tip, the OP polarization would switch to [$001$]. According to our above analysis, the IP polarization would greatly follow the tip's slow scan direction. As the domains merge, DW numbers decrease and the average DW length increases (Fig. 2g and h). Hence, domains with only two polarization directions, i.e. [$111$] and [$\bar{1}11$], are created (Fig. 5c). Here we have T–T DWs pointing along [$010$] randomly located in this area, marked by purple lines. (ii) High-voltage poling. Three regions of about 400 nm in width (marked by red boxes in Fig. 5d), separated by about 800 nm, are polar-scanned with 8 V and the slow scan direction is fixed to [$0\bar{1}0$]. As mentioned above, the electric field from the slow scan direction [$0\bar{1}0$] and the cantilever [$100$] gives [$1\bar{1}0$] IP polarization an advantage over other directions. Meanwhile, under high voltages, T–T DWs have relatively long average lengths and high mobility. Then, as depicted in Fig. 5e, one could naturally expect long tripped domains with [$1\bar{1}1$] polarization after this process, with T–T DWs (purple lines in Fig. 5e) created on the right side of the high-voltage scanned areas.

Indicated by the shattered T–T DWs (bright lines in Fig. 5f), we implemented the above two-step poling in a $2{\rm{\ \mu }}{{\rm{m}}} \times 2{\rm{\ \mu }}{{\rm{m}}}$ area with original mosaic domains, and successfully fabricated parallel linear T–T DWs as conductive paths (bright lines in Fig. 5g). Furthermore, as the scan direction is rotated, conductive paths can be rotated for 90° (see Supplementary Data, Part F).

## DISCUSSION

In summary, we have investigated the dynamics of three types of 71° DWs (H–H, T–T, and H–T) in (001) BFO/SRO/STO films with an applied electric field. Under low voltages, uncharged (H–T) DWs exhibit relatively high mobility, while movements of charged (H–H, T–T) DWs, which have longer total DW lengths, are hindered by charged defects. Under high voltages, the applied electric fields are large enough to provide nucleation energy for charged DWs, and to overcome the pinning effect from the surrounding charged defects, thus contributing to shorter total DW lengths and longer average DW lengths. It is noteworthy that under high voltages the mobility and average DW lengths of conductive T–T DWs are the largest among the three kinds of DWs. Additionally, with increasing applied voltage, IP electric field from the slow scan direction and the cantilever exhibits a stronger influence on the sample, leading to a preferred IP polarization direction and larger average domain size. Based on the above results, we designed a two-step approach incorporating both low- and high-voltage poling processes, and successfully produced arrays of well-aligned and stable conductive T–T DWs. Our work reveals the remarkable impact of charge accumulation around DWs on DW mobility, providing a generalizable approach for DW dynamic studies in ferroic materials. The methodology proposed here for the advanced tunability of conductive DWs makes significant progress towards their applications in functional nano-devices.

## Supplementary Material

nwz176_Supplemental_File
