# Supplementary material for: Dynamics and manipulation of ferroelectric domain walls in bismuth ferrite thin films
Source: Natl Sci Rev. 2019 Nov 8;7(2):278–84. doi: 10.1093/nsr/nwz176 (PMC8288939; doi:10.1093/nsr/nwz176)
Supplement: nwz176_Supplemental_File [file nwz176_supplemental_file.docx]

Supplementary Information

Shuyu Xiao,^1^ Yaming Jin,^1^ Jan-Chi Yang,^2^ Xiaomei Lu,^1,3,a)^ Ying-Hao Chu,^4,b)^ S-W. Cheong,^5^ Jiangyu Li,^6,7^ Yang Li,^1^ Fengzhen Huang,^1,3^ and Jinsong Zhu^1^

**AFFILIATIONS**

^1^National Laboratory of Solid State Microstructures and Physics School, Nanjing University, Nanjing 210093, P. R. China.

^2^Department of Physics, National Cheng Kung University, Tainan 70101, Taiwan.

^3^Collaborative Innovation Center of Advanced Microstructures. Nanjing University, Nanjing 210093, P. R. China.

^4^Department of Materials Science and Engineering, National Chiao Tung University, Hsinchu 30010, Taiwan

^5^Rutgers Center for Emergent Materials and Department of Physics and Astronomy, Rutgers University, Piscataway, New Jersey 08854, USA

^6^Shenzhen Key Laboratory of Nanobiomechanics, Shenzhen Institutes of Advanced Technology, Chinese Academy of Sciences, Shenzhen, Guangdong 518055, China

^7^Department of Mechanical Engineering, University of Washington, Seattle, WA 98195–2600, USA.

**Part A. 3D PFM images**

The IP polarization of the sample is obtained by detecting the twist of the tip, so we can measure IP polarization for only one direction (x or y) at one time. Take the initial domain configuration as an example, as shown in Fig. S1(a), when the SPM tip scans in x direction, only polarizations in y direction could be detected, thus regions with [$\bar{1}\bar{1}0$] and [$110$] IP polarizations can be distinguished as black and white, respectively, while polarizations in x direction, i.e. [$1\bar{1}0$] and [$\bar{1}10$] IP polarizations, are shown in gray. Similar process applies for y direction, as shown in Fig. S1(b), regions with [$1\bar{1}0$] and [$\bar{1}10$] IP polarizations are shown as black and white, respectively, while [$\bar{1}\bar{1}0$] and [$110$] IP polarizations are shown in gray. Then the denoised IP PFM images [Fig. S1(c,d)] and the OP PFM image (monocolor here for the OP polarizations are [$00\bar{1}$] for the whole area) compose a 3D PFM image [Fig. S1(e)].


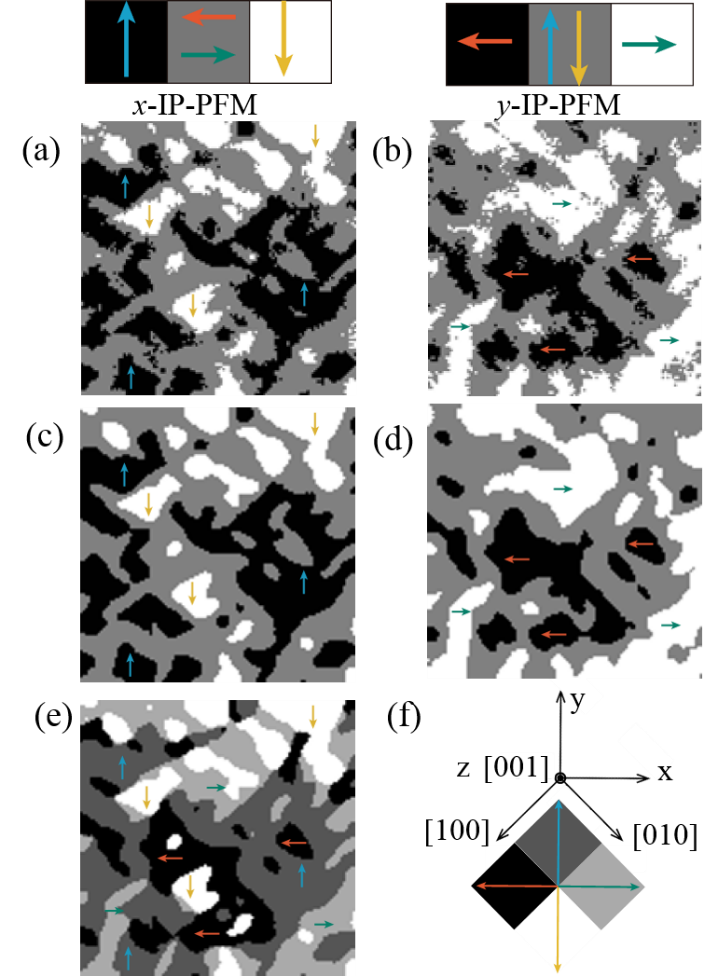


**FIG. S1.** IP polarization combination method of BFO. (a) The original *x*-IP-PFM image. (b) The original *y*-IP-PFM image. (c,d) The denoised version of (a,b). (e) Complete IP polarization image. (f) 4 colorful arrows represent 4 IP polarization directions.

**Part B. IP PFM images corresponding to Fig. 1**

X- and y-direction IP PFM images under various voltages ranging from 0 V to 7.6 V are displayed in Fig. S2. By composing x- and y-direction IP PFM images, as described in part A, complete IP polarization configurations could be generated [Fig. 1].


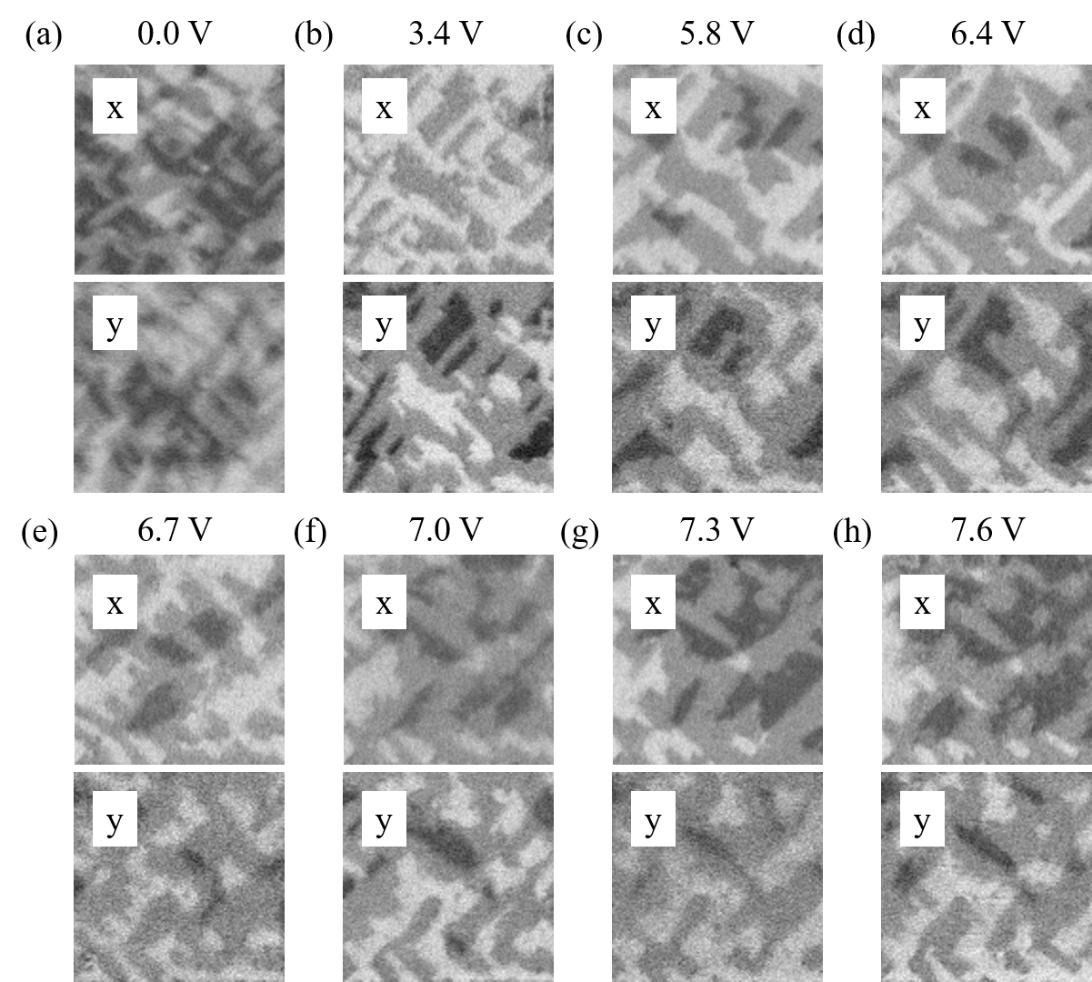


**FIG. S2.** IP PFM images under 0.0 V-7.6 V for the same area of Fig. 1.

**Part C. OP PFM images corresponding to Fig. 1**

As shown in Fig. S3(a), the OP polarization of the as-grown BFO film is in [$00\bar{1}$] direction. After a polar scan with 3.4 V in the red squared area of Fig. S3(a), which is the same area of Fig. 1(a-h), the OP polarization reversed to [$001$] direction [Fig. S3(b)].


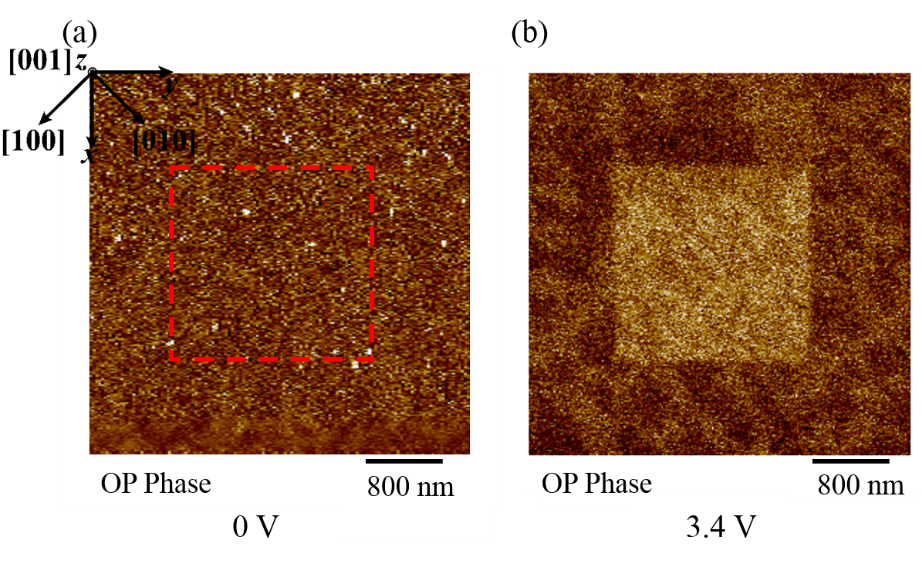


**FIG. S3.** OP PFM images for the (a) initial state and (b) after 3.4 V is applied in the center area.

**Part D. DW movement**

In the maintext, we present how we distinguish 3 kinds of DWs and analyze their dynamics in Fig. 2. Here is another example of such process when the applied voltage increased from 5.8 V to 6.4 V. As shown in Fig. S4(a,b), DW types (H-H, H-T or T-T) are identified manually.


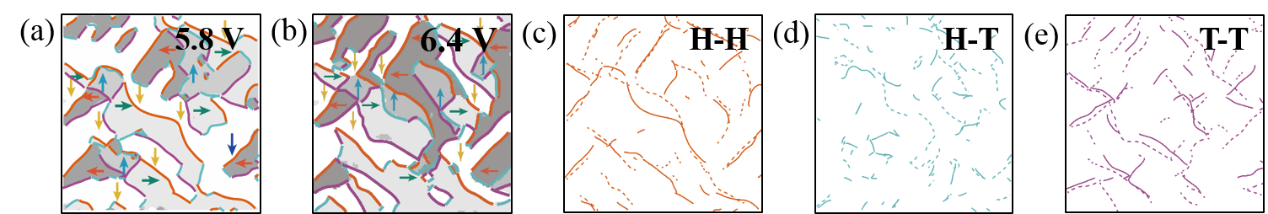


**FIG. S4.** Domain patterns after 5.8 V (a) and 6.4 V (b) poling. Dark, white, light gray, and dark gray area represent domains with polarizations along [$1\bar{1}1$], [$111$], [$\bar{1}11$], and [$\bar{1}\bar{1}1$], respectively. H-H, H-T and T-T DWs are colored by orange, light blue and purple, respectively. (c) H-H, (d) H-T, and (e) T-T DW movements before and after 6.4 V poling. DWs before and after poling in (c-e) are depicted as solid and dashed lines, respectively.

**Part E. Statistics of New DWs**

The numbers of newly created DWs could be roughly found in Fig. 2(g) if we contribute the increase of DW numbers simply to the nucleation of new DWs. To get a clear insight of the DW nucleation, the numbers of new domain walls are depicted in Fig. S5. The trend of new DW numbers for H-H, H-T and T-T DWs under various voltages are quite similar, all exhibiting a peak at the turning point (6.7 V). As the nucleation energy is relatively lower for H-T DWs, compared with H-H and T-T DWs, the new DW numbers of H-T DWs are consistently larger than that of H-H and T-T DWs.





**FIG. S5.** New DW numbers under various voltages.

**Part F. Conductive path manipulation**

As shown in Fig. 3(g), the scheme for conductive path fabrication has been successfully achieved along [$100$] direction. Here we show the scheme is also applicable in [$010$] direction if we rotate the poling scan direction by 90° (Fig. S5).


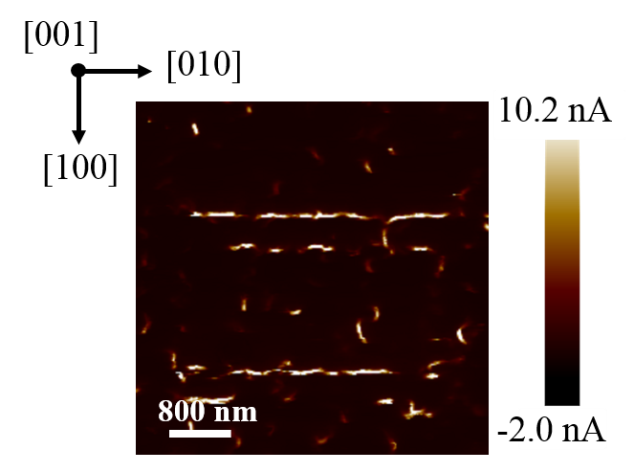


**FIG. S6.** cAFM image showing T-T DWs created along [$010$] direction.
